# A case of chronic myeloid leukaemia presenting as megakaryocytic blast crisis (AML M7)

**DOI:** 10.3332/ecancer.2013.375

**Published:** 2013-11-21

**Authors:** Ponnuswamy Karkuzhali, Velusamy Shanthi, Thiruvengadam Usha

**Affiliations:** 1 Institute of Pathology, Madras Medical College, Chennai, Tamilnadu 600 003, India; 2 Department of Haematology, Madras Medical College, Chennai, Tamilnadu 600 003, India

**Keywords:** chronic myeloid leukaemia, blastic crisis, acute megakaryocytic leukaemia, AML M7

## Abstract

Acute megakaryocytic leukaemia (AMeL) is a rare subtype of acute myeloid leukaemia, which can be frequently misdiagnosed as acute myelofibrosis or myelosclerosis [1]. Chronic myeloid leukaemia (CML) presenting primarily as megakaryocytic blast crisis is very rare, with very few case reports published to date [2, 3]. This case report describes a 36-year-old woman who presented with anaemia and massive splenomegaly with peripheral blood and bone marrow showing features of AMeL. Reverse transcriptase polymerase chain reaction and gel-electrophoretic study of peripheral blood leucocytes demonstrated breakpoint cluster region–Abelson oncogene translocation encoding for p210 fusion protein. Megakaryocytic blast crisis as the primary presentation of CML is very rare and requires clinical correlation and additional cytogenetic studies to determine the diagnosis.

## Introduction

Chronic myeloid leukaemia (CML) is a clonal stem cell disorder, characterised by reciprocal translocation between chromosomes 9 and 22, originally named the ‘Philadelphia chromosome’ (Ph). It has a peak incidence of 53 years, with men outnumbering women. The natural course of CML has three phases: chronic, accelerated, and blast phases. The majority of the patients present in the chronic phase, around 10% present in the accelerated phase, and another 10% in the blast phase. Megakaryocytic blast crisis in patients with CML is unusual, constituting <3% of transformed cases [1].

## Case report

A 36-year-old woman presented with a two-month history of increasing fatigue and abdominal fullness with accompanying loss of appetite. There was no history of fever, jaundice, or bleeding manifestations. There was no history of any antecedent haematological disorder. On examination, the patient had pallor and splenic enlargement, measuring 22.9 cm in ultrasonography. Physical examination was otherwise unremarkable. Complete haemogram findings included haemoglobin of 7.5 g%, red blood cell (RBC) count of 3560 × 10^9^/L, total leucocyte count of 27.9 × 10^9^/L, and a platelet count of 364 × 10^9^/L.

**Figure 1:**
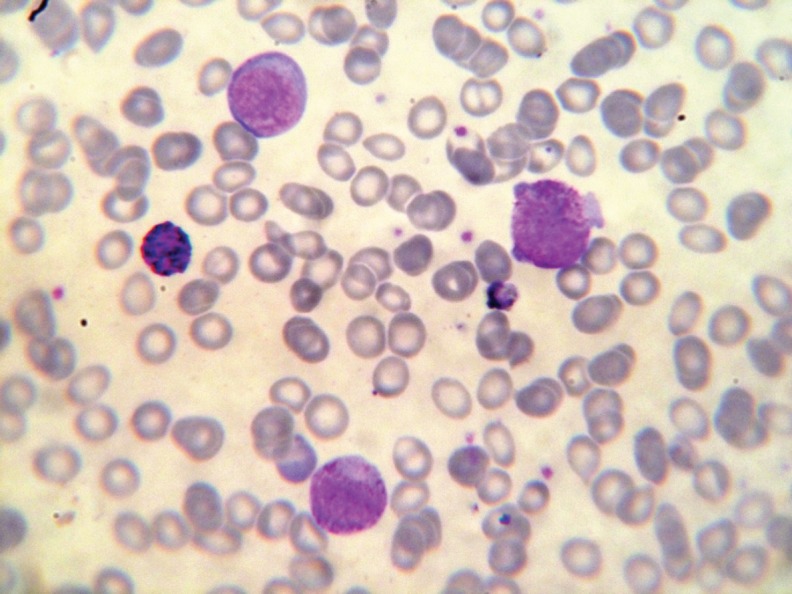
Blasts with cytoplasmic blebs and a basophil.

**Figure 2:**
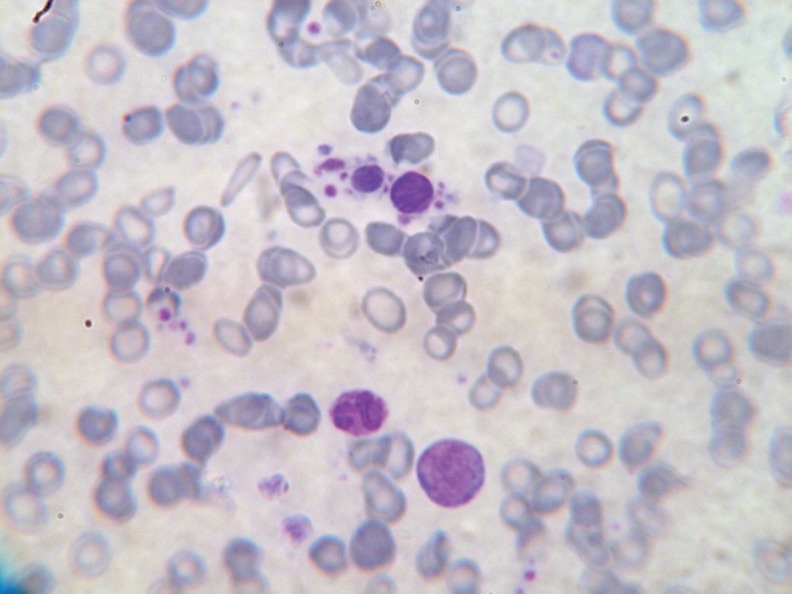
Abnormal, large platelets.

The peripheral smear showed microcytic hypochromic erythrocytes with many dacrocytes, polychromatophilic RBCs, and normoblasts. The leucocyte differential count was blasts 26%, myelocytes 7%, metamyelocytes 6%, basophils 4%, eosinophils 2%, lymphocytes 10%, and neutrophils and band forms 45%. The blasts showed scant basophilic cytoplasm, round nuclei with fine chromatin, and 2–4 nucleoli. Some of the blasts showed cytoplasmic blebs or projections (Figure 1). The neutrophils showed dyspoietic features such as hypo- and hypersegmentation, Pelger–Huet anomaly, and hypogranulation. Many giant platelets and platelet aggregates were seen. Individual platelets showed marked variation in size (Figure 2).

**Figure 3:**
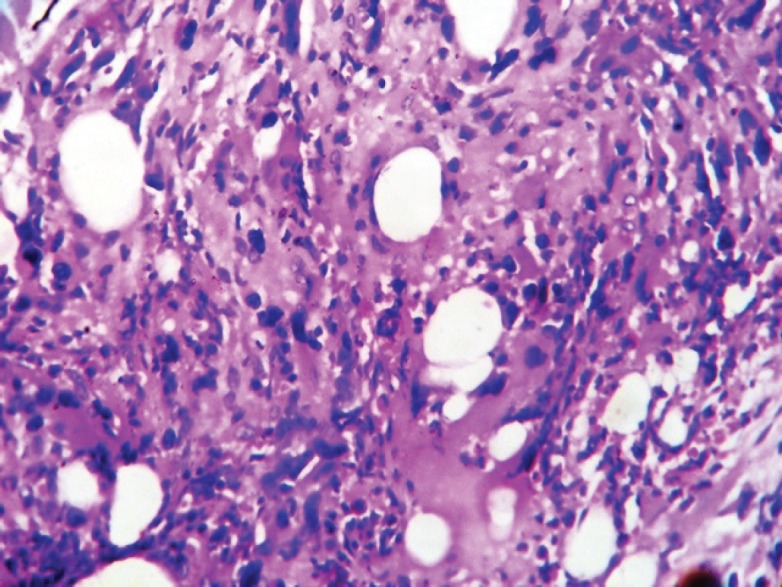
Abnormal megakaryocytes and marrow fibrosis.

**Figure 4:**
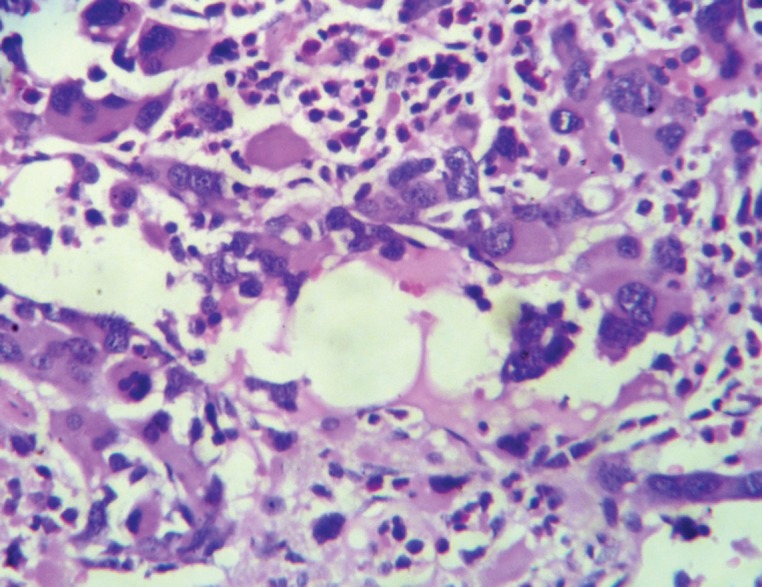
Megakaryocytes with pleomorphic nuclei.

**Figure 5:**
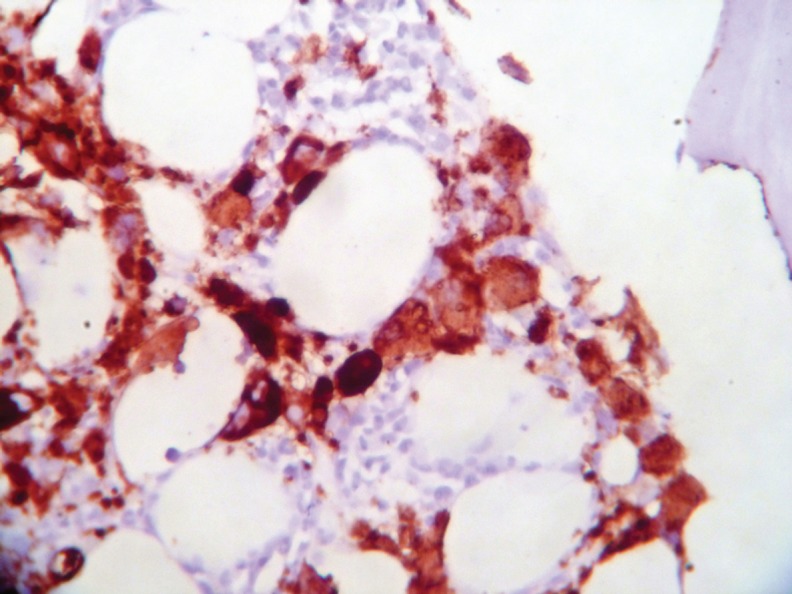
Strong CD 61 positivity in megakaryocytes.

Bone marrow aspiration and biopsy showed increased abnormal megakaryocytes and bone marrow fibrosis (Figure 3). Gomori’s trichrome and reticulin stains revealed a Grade II fibrosis. Monolobated and multinucleated megakaryocytes with hyperchromatic and pleomorphic nuclei were seen (Figure 4) and showed strong positive staining for CD 61 (Figure 5).

A qualitative assay for breakpoint cluster region–Abelson (BCR-ABL) translocation was done on peripheral blood leucocytes using reverse transcriptase polymerase chain reaction and gel electrophoresis. The hybrid transcript for BCR-ABL was detected, and the genomic breakpoint was observed at e14a2 that corresponds to p210.

The patient was started on induction therapy with etoposide, cytarabine, and Adriamycin along with Imatinib Mesylate. She has received two cycles of chemotherapy with the above drugs and was maintained on Imatinib. Her peripheral blood blast count at the end of second cycle was 16%. The patient received chemotherapy again, because of the increased blast cell count. No stem cell transplantation was given due to patient’s non-compliance. No further cytogenetic studies were done to detect additional abnormalities or to assess molecular remission. The patient expired in March 2013 due to blast crisis.

## Discussion

The diagnosis of blast transformation of CML is made by a blast count of >20% in peripheral blood and/or bone marrow, presence of extramedullary blast proliferation or presence of large aggregates of blasts in the marrow. The natural course of transformation from chronic phase to accelerated/blast phase takes four years. Majority of the cases (70%) develop myeloid leukaemia. The diagnosis of acute myeloid leukaemia (AML M7) can be considered when peripheral blood shows blasts with cytoplasmic blebs and platelet abnormalities, and bone marrow shows abnormal megakaryocytes associated with marrow fibrosis. Confirmation of megakaryocytic lineage is by demonstration of megakaryocyte-platelet lineage-specific markers, namely CD41, CD42b, CD61, CD62, and Factor VIII [1]. It is not uncommon to find normal platelet count or thrombocytosis in CML-BC (as in our case) than in *de novo *AML M7. Although difficult to explain, it could be due to megakaryocytic proliferation seen in this condition [2, 5].

Distinguishing *de novo *acute megakaryocytic leukaemia (AMeL) from megakaryocytic blast crisis of CML is important as it has important implications in management of these patients. Although patients with CML present commonly in chronic phase, rarely, they may present in the blast phase, as in our case. The peripheral smear and bone marrow findings are identical in both groups. The findings of massive splenomegaly, basophilia, and thrombocytosis point toward CML. Our patient had massive splenomegaly and basophilia but a normal platelet count. Another distinguishing feature between the two groups is the presence of Ph in patients with CML. However, rare cases of Ph positive *de novo *AML have been reported. These patients do not have additional cytogenetic abnormalities, which can be seen in CML with blast crisis [4].

The prognosis is significantly poor in both *de novo *AML M7 and CML with megakaryocytic blast crisis. AML M7 by itself is an adverse prognostic factor for disease-free survival. However, the treatment of CML patients in blast crisis with a combination of Cytarabine-based induction regimen and the tyrosine kinase inhibitor Imatinib has a significantly better outcome than when treated with induction therapy alone. Also, the initial use of Imatinib helps to revert from blast phase to chronic phase, which can improve the outcome following stem cell transplantation [5].

## Conclusion

AML M7 blastic crisis in CML is a rare manifestation, and the identification of both the conditions is important as the treatment protocols vary in *de novo *AML M7 and AML M7 blastic crisis. In the latter, the initial use of Imatinib followed by combination chemotherapy may improve the outcome and may even convert the blastic crisis of CML into the chronic, stable phase.
